# Copper Sulfate Combined with Photodynamic Therapy Enhances Antifungal Effect by Downregulating AIF1

**DOI:** 10.3390/jof10030213

**Published:** 2024-03-14

**Authors:** Meimei Zhang, Qiyuan An, Yingzhe Wang, Shigan Ye, Xiaoliang Zhu

**Affiliations:** 1 Department of Dermatology, Nanfang Hospital, Southern Medical University, Guangzhou 510515, China; hxz221006@163.com (M.Z.); wangyz412@163.com (Y.W.); y535099213@163.com (S.Y.); 2 Department of Biopharmaceutics, School of Laboratory Medicine and Biotechnology, Southern Medical University, Guangzhou 510515, China; anqiyuanaqy@163.com

**Keywords:** *Candida albicans*, HE-PDT, copper sulfate, oxidative stress

## Abstract

*Candida albicans* is a clinically significant opportunistic fungus that is generally treated with antifungal drugs such as itraconazole and fluconazole. However, the recent emergence of fungal resistance has made treatment increasingly difficult. Therefore, novel antifungal treatment methods are urgently required. Hexanol ethosome photodynamic therapy (HE-PDT) is a method that uses photosensitizers (PS), such as hexanol ethosome, to exert antifungal effects, and can be used to treat resistant fungal strains. However, due to the high dose of PS required for antifungal treatment, excess photosensitizers may remain. Furthermore, once exposed to light, normal tissues or cells are damaged after photodynamic therapy, which limits the clinical application of HE-PDT. Therefore, improving the efficacy without increasing the dose is the key to this treatment. In this study, the antifungal effect of copper sulfate combined with HE-PDT was investigated, and its mechanism was explored. The results suggested that exogenous copper sulfate significantly increased the antifungal effect of HE-PDT by enhancing the rate of *C. albicans* inhibition, increasing reactive oxygen species (ROS) accumulation, increasing the rate of apoptosis, and altering the mitochondrial membrane potential (MMP) and ATP concentration, which is related to the downregulation of apoptosis-inducing factor (AIF1) expression. In conclusion, copper sulfate combined with photodynamic therapy significantly inhibited the activity of *C. albicans* by inducing apoptosis. The combined approach reported herein provides new insights for future antifungal therapy.

## 1. Introduction

Fungal infections are a major global health concern, particularly in immunocompromised populations. On 25 October 2022, the World Health Organization (WHO) first declared *Candida albicans* as an ‘extreme priority’ fungus, calling for strengthening of the global response to fungal infections and antifungal resistance [1]. Because antifungal drugs are widely used, *C. albicans* has evolved in order to survive, leading to more serious drug resistance, making treatment substantially more difficult.

Photodynamic therapy (PDT) is facilitated by the accumulation of photosensitizers (PS), which produce a mass of toxic substances under specific light conditions, such as reactive oxygen species (ROS), which, in turn, cause the apoptosis or necrosis of pathogenic microorganisms [2]. Hexyl-aminolevulinate (HLA) is a photosensitizer and an ester derivative of ALA (5-aminolevulinic acid). It has higher fat solubility and stability than ALA and efficiently produces protoporphyrin IX [3]. Ethosomes (ES) are transdermal drug carriers with a lipid bimolecular vesicle structure; they have good permeability and stability, and can achieve higher permeability for HLA to improve its bioavailability [4]. HLA-ES has achieved significant therapeutic effects in psoriasis, acne, and *C. albicans* biofilms when administered during the early stages [4,5,6], and has obvious advantages over ALA and HLA, which are commonly used in clinics [7]. Although PDT has been advocated for as a therapeutic alternative to antifungal agents [8], high concentrations of PS are associated with significant disadvantages; high PS doses often remain in the body as not all of the compound is used in the reaction. In addition, light exposure causes oxidative damage to normal tissues or cells [9]. Therefore, low-dose PSs that can achieve the ideal therapeutic effects are urgently required.

Copper ions have been previously used to treat fungal infections; over the past few decades, various copper formulations have been used to treat skin diseases, such as eczema, scars, lupus, syphilis, etc. However, the use of copper has been reduced due to the emergence of antibiotics. With the abuse of antibiotics and the emergence of fungal resistance, the role of copper has been re-emphasized [10]. Studies have reported that a combination of copper sulfate and povidone can be used to treat bovine fungal infections and foot rot disease in cattle and sheep, and has an obvious curative effect [11]. Furthermore, the combination of Cu and antifungal drugs has been found to enhance the antifungal effect [12]. The potent triamino acid compound Cu-phendione can inhibit the activity of vaginal *C. albicans* [13]. Cu not only has antibacterial and antifungal effects [14], but also enhances the antifungal activity of combined drugs due to the accumulation of intracellular copper, which induces oxidative stress and disrupts cell function. After copper is transported to the target tissue through its chaperone protein, Cu can catalyze a variety of physiological reactions, including mitochondrial energy production and redox homeostasis. Cu is an important metal element in both prokaryotes and eukaryotes. When in excess, Cu catalyzes the Fenton/Fenton-like reaction, which converts the superoxide anion (O_2_^−^) into a more toxic hydroxyl radical (OH^−^), leading to the accumulation of toxic products in cells and increased cytotoxicity [12].

Because high PS concentrations are required for PDT treatment, a large proportion of the PS remains in the body, which causes oxidative damage to normal tissues or cells [9] when exposed to light. We hypothesized that copper can enhance the antifungal effects of PDT, even at a reduced PS dose. However, studies on the effects of Cu-PDT on fungal infections are scarce. In this study, for the first time, we combined copper sulfate with HE-PDT to evaluate its therapeutic effect against *C. albicans* infections, and to elucidate the underlying mechanisms. Understanding the mechanisms underlying Cu action in cells will provide new insights and may contribute to the development of novel antimicrobial therapies.

## 2. Materials and Methods

### 2.1. Fungal Strains, Growth Conditions, and Agents

*C. albicans* strain SC5314 was obtained from our research group [6,7]. *C. albicans* was cultivated on YPD plates at 37 °C. HLA-ES (HLA: Suzhou Namet Biotechnology Co., Ltd., Suzhou, China) was prepared according to the method described previously [6].

### 2.2. Measurement of Inhibition of C. albicans by HLA-ES

*C. albicans* was adjusted to a concentration of 1 × 10^7^ cells/mL with Roswell Park Memorial Institute 1640 medium (RPMI 1640, Thermo Fisher Scientific, Waltham, MA, USA), mixed with configured HLA-ES (0.0625%, 0.125%), and seeded in a 96-well plate, respectively. It was then wrapped in tin foil and incubated in an incubator at 37 °C for 24 h while protected from light. After sufficient incubation time, the suspension was centrifuged and washed twice with sterile PBS to remove the drug solution, and the cells were resuspended with RPMI 1640 medium and immediately irradiated for 30 min using a light-emitting diode (LED) light source (Wuhan Yage Photonics Co., Ltd., Wuhan, China). Using the serial dilution method, in each group of the suspension, we performed gradient dilution [7], and ultimately, 10 μL of serially diluted cell suspension was coated on the YPD agar plate, subjected to 37 °C incubation for 24 h for colony counting, and we performed three independent experiments.

### 2.3. Inhibition of C. albicans by Copper Sulfate

Copper sulphate solution was first prepared by mixing 2 mL of RPMI 1640 with copper sulphate (Macklin Biochemical Technology Co., Shanghai, China), vortexing to allow full dissolution, and filtering twice through a sterile 0.22 µM filter membrane and setting aside. The RPMI 1640 medium was adjusted with *C. albicans* to a concentration of 1 × 10^7^ cells/mL, which was mixed with copper sulphate (at concentrations of 100, 200, 400, 800, 1600, 3200, 6400, 12,800, 25,600, and 51,200 μM), seeded in 96-well plates, and incubated for 24 h. Groups of each concentration were coated on YPD agar plates using the serial dilution method and placed in an incubator at 37 °C for 24 h. The number of colonies of *C. albicans* was counted and converted to Log logarithm.

### 2.4. Evaluation of the Inhibitory Effect of HE-PDT Combined with Copper Sulfate on C. albicans

The experiments were grouped into four groups: control (*C. albicans*) group, copper sulphate + *C. albicans* group, HE-PDT + *C. albicans* group, and HE-Cu-PDT + *C. albicans* group. The four subgroups were mixed and planted in 96-well plates, respectively, and incubated for 24 h under light protection, and the photodynamic therapy group was washed twice with PBS, and the cells were resuspended in RPMI 1640 medium and immediately irradiated with light-emitting diodes for 30 min. The serial dilution method was adopted to coat all subgroups on YPD agar plates, and they were incubated at 37 °C for 24 h. CFUs were counted and converted to Log logarithm.

### 2.5. Measurement of Intracellular ROS, H_2_O_2_, O_2_^−^, and ATP

Observing ROS: The four subgroups were mixed and planted in 1 mL centrifuge tubes, incubated for 24 h, washed twice with PBS, resuspended in RPMI1640 medium, and irradiated with light-emitting diodes for 30 min; all subgroups were centrifuged and the supernatant was discarded. At the same time, the fluorescent DCFH-DA (2′,7′-Dichlorodihydrofluorescein diacetate) was configured according to a ratio of 1:1000 and mixed well. Then, 100 μL of DCFH-DA was added to each group, gently mixed, put into the incubator at 37 °C to avoid light and incubated for 30 min, centrifuged, and, after we discarded the supernatant, washed with 1 mL of PBS. Then, it was centrifuged again, and, after we discarded the supernatant, resuspended by adding 100 µL of PBS. Then, it was added to a new 96-well plate, and then, observed and photographed under a fluorescence microscope.

Intracellular O_2_^−^ or H_2_O_2_ [15,16,17,18]: After centrifuging and discarding the supernatant of the four treated subgroups, 10 μL of DCFH—DA was added to each of them and the cells were incubated at 37 °C for 30 min, and then, the excess DCFH—DA was washed with PBS. Fluorescence was recorded with a multifunctional microplate reader. For O_2_^−^, the excitation wave was 485 nm and the emission wave was 585 nm. For H_2_O_2_, the excitation wave was 485 nm and the emission wave was 535 nm. The proportions of O_2_^−^ or H_2_O_2_ were calculated using the following formula: [O_2_^−^] or [H_2_O_2_] = average cell strength of the experimental group/the average cell strength of the control group.

Measurement of ATP: After centrifuging and discarding the supernatant of the four treated subgroups, we added 100 μL of working solution to each group, shook and mixed it for 2 min at 25 °C for 10 min, and then, measured the luminescence value of RLU by using the multifunctional enzyme marker.

### 2.6. Flow Cytometry for the Detection of ROS, Apoptosis, and Mitochondrial Membrane Potential (MMP) Changes

Measurement of ROS: The 4 processed subgroups were centrifuged, the supernatant was discarded, and 100 μL DCFH-DA was added to each tube and mixed, incubated for 30 min at 37 °C, protected from light, and washed twice with sterile PBS. The 4 subgroups were resuspended with 300 μL PBS, and the cells were analyzed immediately using a BECKMAN COULTER flow cytometer (CytoFLEX S, Beckman Coulter Company, Brea, CA, USA).

Apoptosis detection: Approximately 100 μL 1 × Annexin V Binding Solution was added to each group. After blowing and mixing, 5 μL FITC conjugate and 5 μL PI were added to the cell suspension and cultured in the dark at 25 °C for 15 min. After adding 400 μL 1 × Binding Solution, the cells were analyzed by flow cytometry.

MMP detection [19]: Firstly, JC-1 was configured into a working solution with RPMI 1640 medium at a concentration of 4 μmol/L according to the instructions. Imaging Buffer (10×) was diluted 10-fold with ultrapure water. A total of 100 μL of the working solution was added into each subgroup and mixed, and then, incubated at 37 °C for 30 min and centrifuged to remove the supernatant. Then, we washed the cells with 200 μL of HBSS twice, and then, centrifuged them to discard the supernatant. The cells were analyzed by a flow cytometer machine immediately after the addition of 200 μL of the 1 × Imaging Buffer Solution.

The data obtained were analyzed by Flojow (FlowJoTM 10.8.1) and independent experiments were repeated at least 3 times.

### 2.7. q-PCR to Verify AIF1 Expression

The yeast RNA rapid extraction kit (Aidlab Biotechnologies Co., Ltd., Beijing, China) was used to collect RNA from each group. 5 × SweScriptAll-in-one SuperMix for qPCR (4 μL), Total RNA (1000 ng/RNA volume obtained previously), and non-enzymatic water were combined, generating a total volume of 20 μL, and then, transcribed into cDNA. cDNA was extracted for the next stage of qPCR using a Bio-Rad IQ5 microplate reader (Thermo Fisher Scientific, Waltham, MA, USA). Amplification specificity was evaluated using a dissolution curve. The transcription level of the ACT1 gene was considered the standardized level of gene expression. Gene expression levels were determined by three independent experiments.

### 2.8. Data Analysis

GraphPad Prism 9 software (San Diego, CA, USA) was used to conduct statistical analysis. A one-way analysis of variance (ANOVA) test was used to evaluate the statistical significance of the differences between the experimental groups and the control group. The data represent the standard deviation of at least three biological replicates. A *p*-value of less than 0.05 was considered statistically significant. * *p* < 0.05, ** *p* < 0.01, *** *p* < 0.001, **** *p* < 0.0001.

## 3. Results

### 3.1. Copper Sulfate Increased the Suppressive Effect of HE-PDT on C. albicans

**Figure 1:**
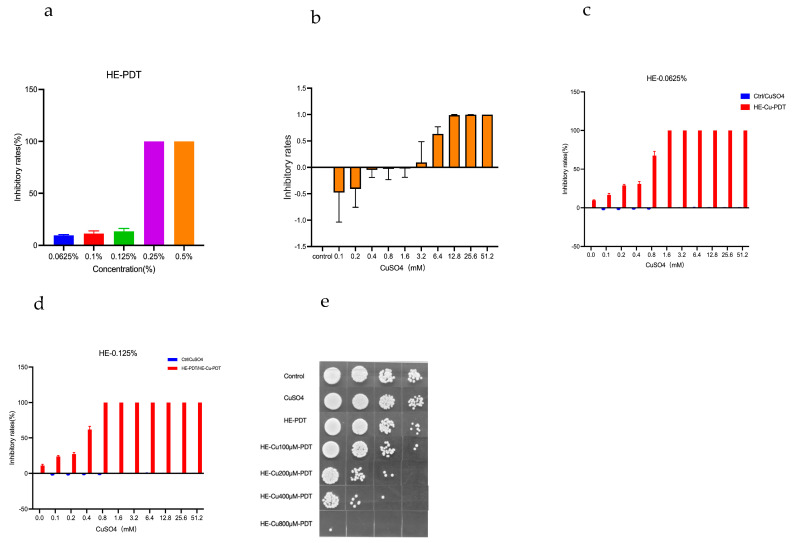
Inhibitory effect of copper sulphate combined with HE—PDT on *C. albicans*. (**a**) HE-PDT inhibition. (**b**) Inhibitory effect of copper sulphate. (**c**,**d**) Comparison of the inhibitory effect of HE (0.0625, 0.125%) concentration synergized with copper sulphate. (**e**) YPD agar plate—number of colony-forming units (CFUs).

The results showed (Figure 1a) that the inhibition of *C. albicans* by 0.25% and 0.5% hexanol plasmapheresis was 100%, whereas the inhibition of *C. albicans* by 0.125% was 13.5%, and the inhibition of *C. albicans* by 0.0625% was 9.633%. Interestingly, copper sulphate had a growth-promoting effect on *C. albicans* when the concentration of copper sulphate was <1.6 mM; however, the inhibition of *C. albicans* was close to 0% at a copper sulphate concentration of 1.6 mM (Figure 1b). At a copper sulfate concentration of 400 μM, HE (0.0625%) inhibited *C. albicans* by 30.81% (Figure 1c); however, at the same concentration of copper sulfate, HE (0.125%) inhibited *C. albicans* by 62.1% (Figure 1d), which boosted the inhibitory rate by half when compared to HE (0.0625%); YPD agar plates (Beijing Wokai Biotechnology Co., Beijing, China) were used to visualize changes in colony numbers (Figure 1e).

### 3.2. Copper Sulfate Enhances Oxidative Stress and Apoptosis

**Figure 2:**
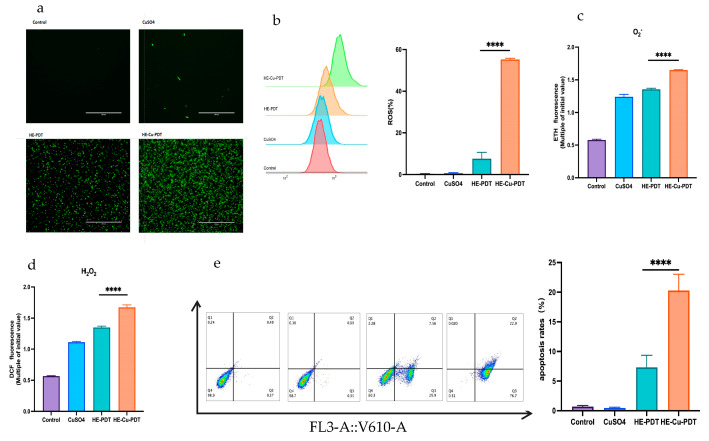
Copper sulphate enhances oxidative stress and apoptosis in *C. albicans* by HE-PDT. (**a**,**b**) HE-Cu-PDT elevates ROS aggregation; (**c**,**d**) HE-Cu-PDT causes an increase in oxidation products; (**e**) HE-Cu-PDT increased the rate of apoptosis. Different colours represent different groups.Each experiment was repeated at least 3 times; **** represents *p* < 0.0001.

Although there was green fluorescence in the HE-PDT group, it was much less than the fluorescence accumulation in the HE-Cu-PDT group (Figure 2a). Meanwhile, in the flow-through results, ROS increased from 7.59% to 55.23% (Figure 2b). The oxidation products H_2_O_2_ and superoxide radicals also accumulate gradually (Figure 2c,d), and PDT acts through cells undergoing apoptosis or necrosis [20,21]; the results showed that the apoptosis rate of HE-PDT was only 7.32%; however, the apoptosis rate was elevated from 7.32% to 20.26% when copper sulphate was combined with HE-PDT (Figure 2e).

### 3.3. Effect of Copper Sulfate on Mitochondria

**Figure 3:**
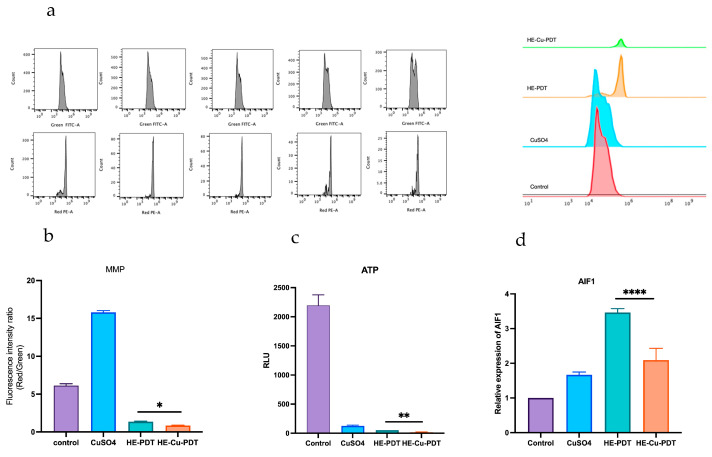
Effect of copper sulfate on MMP and ATP. (**a**,**b**) Mitochondrial membrane potential (MMP) damage detected by JC-1. (**c**) Impaired ATP energy supply. (**d**) The expression of AIF1 was downregulated; Different colours represent different groups. **** represents *p* < 0.0001, ** represents *p* < 0.01, * represents *p* < 0.05.

Mitochondria provide energy for cells and regulate apoptosis [22,23]; it has been shown that HE-Cu-PDT increases the apoptosis rate from 7.32% to 20.26% (Figure 2e). JC-1 acts as a probe, for which the red/green fluorescence ratio is considered to be an assessment of the state of mitochondrial polarization. The experimental results showed that HE-Cu-PDT had a lower wave peak than HE-PDT (Figure 3a) and a decreased red/green fluorescence ratio (Figure 3b). MMP impairment also led to a decrease in ATP energy supply, and the HE-Cu-PDT group presented energy supply values of *C. albicans* that were closer to the horizontal line compared to the HE-PDT group (Figure 3c). Normally AIF1 is an apoptosis-inducing factor located on mitochondria, and the expression of AIF1 was elevated in the HE-PDT group (Figure 3d); however, AIF1 expression was suppressed in the HE-Cu-PDT group.

## 4. Discussion

There is a wide range of antifungal drugs available, such as amphotericin B, fluconazole, echinocandin, and pyrimethamine, often accompanied by side effects such as impaired hepatic and renal function and gastrointestinal tract problems [24]; while resistance is constantly being developed and is accentuated by the prolonged prophylactic use of antifungal drugs [25,26,27,28], the emergence of drug-resistant fungi and the increasing number of high-risk patients has put the inclusion of antifungal resistance on the research agenda of international funding agencies [29].

PDT has been clinically used to treat cancer and viral, bacterial, and fungal infections [30,31,32]; however, its therapeutic effect on fungi is limited, mainly because the passage of PS is hindered by the purine channel in the fungal cell wall. Therefore, a large PS concentration is required to achieve the desired therapeutic effect [32]. Studies have reported that when ALA-PDT is used to treat *C. albicans*, the minimum inhibitory concentration of ALA is 500 mg/mL [33]. However, excessive drug concentrations lead to PS residues remaining in the body, causing damage to tissues and cells [9].

This study demonstrated that the HE-Cu-PDT group significantly increased the inhibition rate of *C. albicans* from 13.5% to 62.1% compared with the HE-PDT group, and this inhibition rate was not a simple two-by-two addition, but reflected a synergistic effect. Some studies have reported that the mechanism by which PDT causes cell death includes apoptosis or necrosis [20,21]. Relevant studies reported that the apoptosis rate of ALA-PDT against *C. albicans* was only 19.4% at 15 mM [34]. However, the findings in this paper showed that the HE-Cu-PDT group significantly increased the apoptosis rate of *C. albicans* from 7.32% to 20.26% compared with the HE-PDT group (Figure 2e); similar synergistic effects have been more extensively studied in tumor and bacterial treatments, especially in tumors, whereas they are rarely seen in antimicrobial treatments, which makes the study of the effect of HE-Cu-PDT on fungus somewhat innovative [35,36,37,38,39,40].

Studies have shown that mitochondria play a role in the efficacy of PDT [22,23]. Our experimental results confirmed that the effect of HE-Cu-PDT is related to mitochondria. The addition of copper sulfate can lead to changes in MMP, ATP concentration, and AIF1 expression. The main reason for this is that copper can lead to mitochondrial oxidative stress [41,42,43], and the cytotoxicity of copper is closely related to oxidative stress. O_2_^−^ and Cu^2+^ react to generate O_2_ and Cu^1+^, providing oxygen for PDT. In addition, Cu^1+^ can react with hydrogen peroxide produced by PDT to form hydroxyl radicals [12], and hydroxyl radicals (OH^−^) are one of the strongest oxidants described thus far [44,45].

It has been reported that apoptosis-inducing factor (AIF1), as a member of the mitochondrial respiratory electron transport chain, has the biochemical nature of NADH dehydrogenase, which is able to promote mitochondrial respiratory function [46,47]; when it is inhibited, electron transport is blocked, resulting in downstream reactions that cannot proceed normally, and impaired ATP production will result in insufficient energy supply for *C. albicans* growth, thus accelerating *C. albicans* apoptosis. So, is it possible that *C. albicans* fails to grow during this process because of respiration inhibition? The synergistic effect of copper sulphate and HE-PDT not only reduces mitochondrial membrane potential, but also leads to a decrease in the production of ATP, which are the main indicators for detecting the function of mitochondria; *C. albicans* was inhibited, probably because of damage to mitochondria, which blocked electron transfer, affecting its respiratory function, and ultimately failed to grow; the specific mechanism of its death deserves further exploration.

In the clinic, there have been different clinical strains of *C. albicans*, such as ATCC 24433 and 90029. However, the *C. albicans* strain SC5314, as a standard strain, was used in this study, and our results showed that photodynamic therapy combined with copper sulfate will provide a new therapeutic idea for the current issue of fungal drug resistance.

## Data Availability

Data are contained within the article.

## References

[1] World Health Organization WHO Fungal Priority Pathogens List to Guide Research, Development and Public Health Action. WHO. https://www.who.int/publications/i/item/9789240060241.

[2] Kwiatkowski S., Knap B., Przystupski D., Saczko J., Kędzierska E., Knap-Czop K., Kotlińska J., Michel O., Kotowski K., Kulbacka J. (2018). Photodynamic therapy—Mechanisms, photosensitizers and combinations. Biomed. Pharmacother..

[3] Morrow D.I., McCarron P.A., Woolfson A.D., Juzenas P., Juzeniene A., Iani V., Moan J., Donnelly R.F. (2010). Hexyl aminolaevulinate is a more effective topical photosensitiser precursor than methyl aminolaevulinate and 5-aminolaevulinic acids when applied in equimolar doses. J. Pharm. Sci..

[4] Yang L., Wu L., Wu D., Shi D., Wang T., Zhu X. (2017). Mechanism of transdermal permeation promotion of lipophilic drugs by ethosomes. Int. J. Nanomed..

[5] Wang T., Wu L., Wang Y., Song J., Zhang F., Zhu X. (2022). Hexyl-aminolevulinate ethosome–mediated photodynamic therapy against acne: In vitro and in vivo analyses. Drug Deliv. Transl. Res..

[6] Wang Y., Song J., Zhang F., Zeng K., Zhu X. (2020). Antifungal Photodynamic Activity of Hexyl-Aminolevulinate Ethosomes Against Candida albicans Biofilm. Front. Microbiol..

[7] Wang Y., Long W., Zhang F., Zhang M., Zeng K., Zhu X. (2022). Hexyl-Aminolevulinate Ethosomes: A Novel Antibiofilm Agent Targeting Zinc Homeostasis in Candida albicans. Microbiol. Spectr..

[8] Nunes I.P., Crugeira P.J., Sampaio F.J., de Oliveira S.C., Azevedo J.M., Santos C.L., Soares L.G., Samuel I.D., Persheyev S., de Ameida P.F. (2023). Evaluation of dual application of photodynamic therapy—PDT in Candida albicans. Photodiagn. Photodyn. Ther..

[9] Yuan B., Wu H., Wang H., Tang B., Xu J.F., Zhang X. (2021). A Self-Degradable Supramolecular Photosensitizer with High Photodynamic Therapeutic Efficiency and Improved Safety. Angew. Chem. Int. Ed. Engl..

[10] Grass G., Rensing C., Solioz M. (2011). Metallic copper as an antimicrobial surface. Appl. Environ. Microbiol..

[11] Zhang J., Wang L., Qin Y., Zhang L., Jiang Y., Cao Y. (2017). Regulation and control of transcription factor *Cup2* on Cu^2+^ metabolism and oxidative stress in *Candida albicans*. J. Pharm. Pract..

[12] Chudzik B., Tracz I.B., Czernel G., Fiołka M.J., Borsuk G., Gagoś M. (2013). Amphotericin B–copper(II) complex as a potential agent with higher antifungal activity against Candida albicans. Eur. J. Pharm. Sci..

[13] Rigo G.V., Cardoso F.G., Devereux M., McCann M., Macedo A.J., Santos A.L.S., Tasca T. (2023). Antimicrobial and Antibiofilm Activities of Copper(II)-1,10-phenanthroline-5,6-pione Against Commensal Bacteria and Fungi Responsible for Vaginal Microbiota Dysbiosis. Curr. Microbiol..

[14] Li C., Li Y., Ding C. (2019). The Role of Copper Homeostasis at the Host-Pathogen Axis: From Bacteria to Fungi. Int. J. Mol. Sci..

[15] Lin C., Kang J., Zheng R. (2005). Oxidative stress is involved in inhibition of copper on histone acetylation in cells. Chem. Interact..

[16] Miller F.J., Gutterman D.D., Rios C.D., Heistad D.D., Davidson B.L. (1998). Superoxide production in vascular smooth muscle contributes to oxidative stress and impaired relaxation in atherosclerosis. Circ. Res..

[17] Somers M.J., Mavromatis K., Galis Z.S., Harrison D.G. (2000). Vascular superoxide production and vasomotor function in hypertension induced by deoxycorticosterone acetate–salt. Circ..

[18] LeBel C.P., Ischiropoulos H., Bondy S.C. (1992). Evaluation of the probe 2′,7′-dichlorofluorescin as an indicator of reactive oxygen species formation and oxidative stress. Chem. Res. Toxicol..

[19] Hamuro J., Yamashita T., Otsuki Y., Hiramoto N., Adachi M., Miyatani T., Tanaka H., Ueno M., Kinoshita S., Sotozono C. (2023). Spatiotemporal Coordination of RPE Cell Quality by Extracellular Vesicle miR-494-3p Via Competitive Interplays with SIRT3 or PTEN. Investig. Opthalmol. Vis. Sci..

[20] Kessel D. (2019). Apoptosis, Paraptosis and Autophagy: Death and Survival Pathways Associated with Photodynamic Therapy. Photochem. Photobiol..

[21] Gheewala T., Skwor T., Munirathinam G. (2017). Photosensitizers in prostate cancer therapy. Oncotarget.

[22] Calixto G.M.F., Bernegossi J., De Freitas L.M., Fontana C.R., Chorilli M., Grumezescu A.M. (2016). Nanotechnology-Based Drug Delivery Systems for Photodynamic Therapy of Cancer: A Review. Molecules.

[23] Yaqoob M.D., Xu L., Li C., Leong M.M.L., Xu D.D. (2022). Targeting mitochondria for cancer photodynamic therapy. Photodiagn. Photodyn. Ther..

[24] Chen Q. (2012). Rational application of antifungal drugs in the treatment of superficial cutaneous fungal diseases. China Med. Guide.

[25] d’Enfert C., Kaune A.K., Alaban L.R., Chakraborty S., Cole N., Delavy M., Kosmala D., Marsaux B., Frois-Martins R., Morelli M. (2021). The impact of the Fungus-Host-Microbiota interplay upon Candida albicans infections: Current knowledge and new perspectives. FEMS Microbiol. Rev..

[26] Brown G.D., Denning D.W., Gow N.A.R., Levitz S.M., Netea M.G., White T.C. (2012). Hidden killers: Human fungal infections. Sci. Transl. Med..

[27] Peacock J.E., Morris A.J., Tanner D.C., Nguyen M.L., Snydman D.R., Wagener M.M., Rinaldi M.G., Yu V.L. (1996). The changing face of candidemia: Emergence of non-Candida albicans species and antifungal resistance. Am. J. Med..

[28] Chowdhary A., Sharma C., Meis J.F. (2017). Candida auris: A rapidly emerging cause of hospital-acquired multidrug-resistant fungal infections globally. PLoS Pathog..

[29] Fisher M.C., Alastruey-Izquierdo A., Berman J., Bicanic T., Bignell E.M., Bowyer P., Bromley M., Brüggemann R., Garber G., Cornely O.A. (2022). Tackling the emerging threat of antifungal resistance to human health. Nat. Rev. Microbiol..

[30] Smijs T.G., van der Haas R.N., Lugtenburg J., Liu Y., de Jong R.L., Schuitmaker H.J. (2004). Photodynamic treatment of the dermatophyte Trichophyton rubrum and its microconidia with porphyrin photosensitizers. Photochem. Photobiol..

[31] Smijs T.G.M., Bouwstra J.A., Schuitmaker H.J., Talebi M., Pavel S. (2007). A novel ex vivo skin model to study the susceptibility of the dermatophyte Trichophyton rubrum to photodynamic treatment in different growth phases. J. Antimicrob. Chemother..

[32] Rodríguez-Cerdeira C., Martínez-Herrera E., Fabbrocini G., Sanchez-Blanco B., López-Barcenas A., El-Samahy M., Juárez-Durán E.R., González-Cespón J.L. (2021). New Applications of Photodynamic Therapy in the Management of Candidiasis. J. Fungi.

[33] Liang Y., Lu L., Chen Y., Yin Q. (2013). Inhibitory effect of 5-aminolevulinic acid photodynamic therapy on Candida albicans. Hainan Med..

[34] Shi H., Li J., Peng C., Xu B., Sun H. (2021). The inhibitory activity of 5-aminolevulinic acid photodynamic therapy (ALA-PDT) on Candida albicans biofilms. Photodiagn. Photodyn. Ther..

[35] Tang H.-X., Liu C.-G., Zhang J.-T., Zheng X., Yang D.-Y., Kankala R.K., Wang S.-B., Chen A.-Z. (2020). Biodegradable Quantum Composites for Synergistic Photothermal Therapy and Copper-Enhanced Chemotherapy. ACS Appl. Mater. Interfaces.

[36] Wang D., Wu H., Lim W.Q., Phua S.Z.F., Xu P., Chen Q., Guo Z., Zhao Y. (2019). A Mesoporous Nanoenzyme Derived from Metal–Organic Frameworks with Endogenous Oxygen Generation to Alleviate Tumor Hypoxia for Significantly Enhanced Photodynamic Therapy. Adv. Mater..

[37] Liang X., Chen M., Bhattarai P., Hameed S., Tang Y., Dai Z. (2021). Complementing Cancer Photodynamic Therapy with Ferroptosis through Iron Oxide Loaded Porphyrin-Grafted Lipid Nanoparticles. ACS Nano.

[38] Cui Y., Chen X., Cheng Y., Lu X., Meng J., Chen Z., Li M., Lin C., Wang Y., Yang J. (2021). CuWO_4_ Nanodots for NIR-Induced Photodynamic and Chemodynamic Synergistic Therapy. ACS Appl. Mater. Interfaces.

[39] Nain A., Tseng Y.-T., Wei S.-C., Periasamy A.P., Huang C.-C., Tseng F.-G., Chang H.-T. (2020). Capping 1,3-propanedithiol to boost the antibacterial activity of protein-templated copper nanoclusters. J. Hazard. Mater..

[40] Lin L.S., Song J., Song L., Ke K., Liu Y., Zhou Z., Shen Z., Li J., Yang Z., Tang W. (2018). Simultaneous Fenton-like Ion Delivery and Glutathione Depletion by MnO_2_—Based Nanoagent to Enhance Chemodynamic Therapy. Angew. Chem. Int. Ed. Engl..

[41] Chen Z., Wu Y., Yao Z., Su J., Wang Z., Xia H., Liu S. (2022). 2D Copper(II) Metalated Metal–Organic Framework Nanocomplexes for Dual-enhanced Photodynamic Therapy and Amplified Antitumor Immunity. ACS Appl. Mater. Interfaces.

[42] Gaetke L.M., Chow C.K. (2003). Copper toxicity, oxidative stress, and antioxidant nutrients. Toxicology.

[43] Theophanides T., Anastassopoulou J. (2002). Copper and carcinogenesis. Crit. Rev. Oncol. Hematol..

[44] Tong M., Yuan S., Ma S., Jin M., Liu D., Cheng D., Liu X., Gan Y., Wang Y. (2016). Production of Abundant Hydroxyl Radicals from Oxygenation of Subsurface Sediments. Environ. Sci. Technol..

[45] Pan J., Deng J., Zhang Q., Yan Q. (2019). A Review of the Application of Advanced Oxidation Technology of Hydroxyl Radicals. J. Guangdong Univ. Technol..

[46] Herrmann J.M., Riemer J. (2020). Apoptosis inducing factor and mitochondrial NADH dehydrogenases: Redox-controlled gear boxes to switch between mitochondrial biogenesis and cell death. Biol. Chem..

[47] Liang M., Huang L., Liu Q., Long R., Deng Y. (2021). Potential ferroptosis pathway in pathogenic fungi: Reported functions and future perspectives. Microbiol. Bull..

